# 

**Published:** 1909-04-15

**Authors:** 


### ANNOUNCEMENTS.

MICHIGAN STATE DENTAL SOCIETY. The fifty-third annual convention of the Michigan State Dental Society will be held at Kalamazoo on June 29th, 30th and July 1st. An interesting and profitable program is now in course of preparation, and a most helpful and enjoyable meeting is assured.

J. W. LYONS, *President.*
DON. M. GRAHAM, *Secretary.*

THE CALIFORNIA STATE DENTAL ASSOCIATION and the Alumni Association, College of Dentistry, University of California, will hold a joint meeting on July 6, 7, 8 at the College Building 2nd and Parnassus Aves., San Francisco.

Arrangements are being made for a number of prominent eastern dentists to be present and contribute to the clinics and papers in addition to members from the State.

Manufacturers are being solicited to make exhibits and in as much as there will be a series of meetings on the Coast from June 28th to July 23rd, it is expected that exhibitors will find it to their advantage to make the circuit.

Fuller details of program will be announced next.

---

THE PENNSYLVANIA BOARD OF DENTAL EXAMINERS will conduct examinations simultaneously in Philadelphia and Pittsburgh, June 9th, 10th, 11th and 12th, 1909.

For application papers, or any other information, write to Dr. Nathan C. Schaeffer, Secretary, Dental Council, Harrisburg, Pennsylvania.

Very respectfully,

W. D. DE LONG, *Secretary.*

---

ILLINOIS STATE DENTAL SOCIETY, will hold its next meeting at Danville, May 11th to 14th.

---

KENTUCKY STATE DENTAL ASSOCIATION will hold its next annual meeting at Crab Orchard Springs, May 17th to 19th. The profession generally is invited as a good program and pleasant meeting place are promised.

---

OHIO BOARD OF EXAMINER'S MEETING. The regular spring meeting of the Ohio State Dental Board will be held in the city of Columbus on June 15 to 18, 1909, for the examination of applicants for license.

All persons desiring to enter practice in this State must make written application for examination.

Applications must be in the hands of the Secretary not later than June 25th, together with the fee of $25.00.

For further information and blank applications address:

F. R. CHAPMAN, *Secretary*,
305 Schultz Building,
Columbus, Ohio.

---

TENNESSEE STATE DENTAL ASSOCIATION meets in Memphis, May 25th to 27th.

---

FOLRIDA STATE DENTAL SOCIETY. The twenty-sixth annual meeting of the Florida State Dental Society will be held in Ocala, Thursday, June 17th, 1909, continuing in session three days.

A cordial invitation is extended to ethical practitioners.

CARROLL H. FRANK, *Secretary*,
301-2 Masonic Temple,
Jacksonville, Florida.

---

COMPLIMENTARY BANQUET TO DR. C. R. BUTLER OF CLEVELAND. On the Eleventh of March there assembled at the Hollenden hotel in Cleveland about two hundred dentists to give expression of the esteem in which they held Dr. Butler for his long and admirable career as a dentist and friend. There were representatives from several cities outside of Ohio and Cleveland and one of ehe handsomest tables we have ever seen was provided. It was a genuine love feast and one that everyone seemed to enjoy. Dr. Butler began practice in Cleveland in 1855, he graduated from the Pennsylvania Dental College in 1858, and the Cleveland Medical College in 1865, he was Professor of Clinical Operative Dentistry in the Ohio Dental College in 1866 and 67, President Northern Ohio Dental Association in 1867, Ohio State Dental Society 1874, American Dental Association 1889, Member Ohio State Board of Dental Examiners 1874 to 1891, and President Celveland Dental Society 1896. He has had a long and successful career as a practitioner of dentistry and the people of Cleveland are very largely indebted to him for his high grade ministrations through all these years, and also for the influence he has always exerted to bring his associates in practice up to the highest standard of efficiency. One can hardly value the long and useful career of such a man and every community that has had the good fortune to have such an one give his long and talented life to it in such a way has a debt of obligation that is seldom thought of or appreciated. The following toasts were given, and the Cleveland friends gave to Dr. Butler a luxurious arm chair and conferred upon him the title of Dean of the Dentists of Northern Ohio. Toast Master: Dr. W. T. Jackman, Cleveland; "Dr. Butler and the National Dental Association." Dr. Chas. S. Butler, Buffalo, N. Y.; "Dr. Butler and the Ohio State Dental Society," Dr. H. C. Brown, Columbus, Ohio; "Dr. Butler and the Northern Ohio Dental Association," Dr. L. L. Barber, Toledo, Ohio; "Dr. Butler and the Cleveland Dental Society," Dr. H. S. Barnes, Cleveland, Ohio; "Dr. Butler and the State Board of Dental Examiners," Dr. J. A. Libbey, Pittsburgh, Pa.; "Dr. Butler and Dental Education," Dr. N. S. Hoff, Ann Arbor, Mich.; "Dr. Butler and Dental Literature," Dr. L. P. Bethel, Columbus, Ohio; "Dr. Butler and Dental Inventions," Dr. H. L. Ambler, Cleveland, Ohio; "Dr. Butler and his Friends," Dr. W. H. Whitslar, Cleveland, Ohio; "The Dental Profession as I have known it," Dr. Chas. R. Butler, Cleveland, Ohio.

